# Seven Shades of Black Thoughts: COVID-19 and Its Psychological Consequences on Cancer Patients

**DOI:** 10.3389/fonc.2020.01357

**Published:** 2020-07-10

**Authors:** Mattia Garutti, Francesco Cortiula, Fabio Puglisi

**Affiliations:** ^1^Dipartimento di Oncologia Medica, Centro di Riferimento Oncologico di Aviano, IRCCS, Aviano, Italy; ^2^Department of Medicine, University of Udine, Udine, Italy

**Keywords:** SARS-COV-2, COVID-19, quarantine, cancer, psycho-oncology, loneliness

## Abstract

An outbreak of novel coronavirus disease (COVID-19) that started in China at the end of 2019 has rapidly spread all over the world. COVID-19 is plaguing people not only physically but also psychologically, and cancer patients are particularly exposed to this emotional threat. Herein, we describe the psychological threats posed by COVID-19 to cancer patients. Our analysis is based on the concerns of our patients during our daily clinical interactions in both outpatient and inpatient settings. We have summarized the patients' psychological issues: logistic overload, loneliness, fear, oxymoronic thoughts, helplessness, frustration, and emotional siege. We describe these psychological threats, provide clinical context for them, and offer practical suggestions for managing them, for the benefit of patients, their caregivers, and clinicians. Our hope is that, by sharing our clinical experience, we can help other oncologists increase their awareness of the psychological impact of the pandemic on cancer patients and implement solutions. Managing these challenges now should translate into improved standards of care when this infective storm is over. Paradoxically, COVID-19 could be an opportunity to learn how to better manage cancer care.

## Introduction

Taking care of the body is easy; taking care of the spirit is really hard (Chinese proverb). COVID-19 is plaguing people around the world not only physically but also psychologically, and cancer patients are particularly exposed to this emotional threat. In oncology, this threat has a practical meaning since patients' perceptions have been shown to not only affect their quality of life but also to stimulate the onset of symptoms (1). In addition, when a patient is approaching the end of his or her life, every single day has special meaning. The obligation to live an aseptic life could have detrimental consequences.

The SARS-COV-2 pandemic has challenged not only governments, institutions, and researchers, but also doctors in new and unexpected ways (2, 3). Managing all these challenges effectively will likely improve the standards of care even when this infective storm is over. Paradoxically, COVID-19 could be an opportunity to learn how to manage cancer care more effectively. In this essay, we discuss the psychological threats the pandemic poses to cancer patients and the concerns our patients spontaneously expressed during routine outpatient and inpatient visits, without formal interviews or the administration of questionnaires.

What are the psychological “enemies” that cancer patients have to live with in these times? We have observed several during our daily clinical practice, and we have summarized them in seven issues: logistic overload, loneliness, fear, oxymoronic thoughts, helplessness, frustration, and emotional siege. Here we describe these issues and provide suggestions to manage them. Our hope is that, by sharing our clinical experiences, we can help other oncologists increase their awareness of the psychological impact of the pandemic on cancer patients and implement some solutions.

## The Seven Shades of Black Thoughts

### Logistic Overload

Due to policies for infection prevention, relatives, friends, and caregivers are prohibited from entering the hospital, and patients may go alone to their medical appointments. Lack of social support and the need to take full responsibility can feel like an information overload for patients, and can result in doubt, decisional paralysis, and low compliance (4–6).

### Loneliness

To limit the spread of the virus, social proximity, and physical contact—including that with physicians—have been prohibited. In addition, the requirement to wear a mask instantly erases a fundamental human connector: the smile. These factors are communication barriers that spoil the empathic channel between healthcare staff and patients. A deep sense of loneliness is the consequence. This loneliness can extend outside the hospital: after returning home, patients face isolation and the same preventive measures already seen in the medical care setting. Loneliness for a cancer patient seems to never end. Indeed, the end of life can be pervaded by feelings of exclusion: a terminally ill patient with COVID-19 has to be isolated, so caresses can only be given through gloves. Loneliness can deeply impair quality of life (7) and can also trigger the onset of symptoms (1).

### Fear

Fear can escalate quickly because of the continuous, redundant coverage of the SARS-CoV-2 outbreak by the mass media (8). Since cancer patients may be prone to severe COVID-19 (9), their fears could be refractory to reassurance. Patients may also fear that doctors are distracted by the emergency, leading them to provide suboptimal cancer care (10). Moreover, cancer patients could be concerned about being a source of contagion for those who take care of them.

### Oxymoronic Thoughts

Patients can have conflicting thoughts pervaded by internal contrasts, when an idea and its opposite are both true in the same instance. This phenomenon can undermine patients' certainties. For instance, hospitals represent the place where patients get medical care but can also get infections. Additionally, cancer treatments are perceived not only as the only hope to fight cancer but also a threat for the immune system that is supposed to protect the patient against infection. Even relatives and caregivers can have oxymoronic thoughts: family members help patients during cancer treatment, but at the same time can be a virus carrier.

### Helplessness

Since the life of a cancer patient is at stake, feeling helplessness can be particularly devastating. Moreover, helplessness can create a conflict between expectations and reality, weakening a patient's trust in physicians and worsening the sense of loneliness.

### Frustration

Many cancer patients know that their life is at risk. This awareness can push them to seek gratification in the present rather than in the future. Unfortunately, quarantine and exceptional measures put in place to limit the spread of the virus can disrupt many moments of pleasure. The desire for joy collides with notices of “closed due to pandemic” displayed on many storefronts.

### Emotional Siege

These days, many cancer patients face bad news from two areas: oncology and the pandemic. When multiple adverse circumstances stack up, the perception of fighting a battle that is impossible to win increases. This perception can trigger a complex, dynamic range of emotions such as anger, sadness, and depression. These emotions have been described by Kübler-Ross in 1970 (11) and have been revised by others more recently (12, 13).

## Discussion

We are well aware that no instrument or tool can substitute interpersonal relationships and the closeness and affection of loved ones. Nonetheless, there is much room for improving patients' emotive health while minimizing the impact on therapeutic outcomes (1, 14). Online and telematics services have been tested and shown to be effective in providing psychological and mental health assistance (15, 16). The key point is how to integrate them into patient care.

Clinicians and healthcare workers—the only human relations for some cancer patients in times of quarantine—should step up and somehow compensate for the psychological support that a caregiver normally provides. This means a better recognition of the psychological processes in each patient, to open a personalized empathetic channel that culminates in a satisfying moment for both patients and healthcare workers. From a pragmatic point of view, this could be realized by dedicating more time during visits to explaining choices and talking about patients' feelings. Especially after a first oncologic visit, patients need time to elaborate the information received and to formulate questions (17). Thus, a scheduled follow-up with the physician via phone or video call may be useful. This is the right moment to bring the communication to the next level, to relieve patients of their negative thoughts and to encourage treatment compliance. For this process to be effective, clinicians should receive focused, albeit not too time-consuming, psychological training, should have a team of psychologists to work with, and should be supported with the necessary technological tools.

The support of a psycho-oncologist is fundamental to help clinicians recognize patients' emotions and psychological mechanisms, and therefore be able to comfort patients with the right words (18). Investing more time in talking with patients may be counterintuitive during a time of crisis, when resources are already stretched, but it may ultimately save time: fewer adverse events would occur because patients understand how to take medications, and there would be less need for treatment adjustments and unscheduled appointments.

Further improvement may be achieved by scheduling telematics contact with the psycho-oncology team, especially from the beginning of the care pathway. An initial online survey may identify the most urgent cases and delineate individual patient's needs, helping to tailor subsequent psycho-oncology interventions. This strategy has been already proposed for the prevention of suicide (19). Elderly patients may have difficulty using digital tools, so an alternative telephonic system should also be available.

Giving cancer patients a care plan as detailed as possible, together with supplementary informative material, is fundamental to compensate for the absence of caregivers' logistic support and to help patients remember all the information received during the first visit. Providing contact information (e.g., e-mail address or phone number) of the doctor or referral team should lower the anxiety of not having a reference point or a way to get in touch when needed. During a pandemic, only necessary hospital admissions are allowed, so follow-up via phone calls should be done whenever possible. At the same time, preserving visual contact through video calls should avoid impersonal patient management and help clinicians evaluate clinical signs (e.g., adverse effects such as skin rash or hand-foot syndrome).

End of life loneliness requires special consideration. Every effort should be made to facilitate communication between patients and their loved ones (e.g., through the supply of smartphones or tablets and by providing assistance in making video calls). Providing psychological support to relatives, in order to relieve potential guilt for not being present, is also important. Caregivers should receive psychologic counseling to help them with the challenges of digital communication. Telematics support items should be described, categorized, and evaluated, like the other cancer care items (20). Periodic reports are also needed to make adjustments accordingly. Telephone and video consultations and psychological support, however, are not intended to substitute for personal relationships among individuals and between patients and their doctors, which constitute the true and unreplaceable support network for cancer patients.

Integrating all these strategies in clinical practice during the pandemic could be challenging and it should be a gradual process. We believe that telemedicine for psychological consultations should be the first step to develop, for both scheduled appointments and emergencies. Not forgetting the emotional sphere of cancer patients in a time of crisis will translate into better care for our patients in the future, and may teach doctors to be more sensitive to these themes. Furthermore, developing a telematics support system now means having it ready whenever needed in the future.

## Data Availability Statement

The original contributions presented in the study are included in the article/supplementary material, further inquiries can be directed to the corresponding author/s.

## Author Contributions

All authors listed have made a substantial, direct and intellectual contribution to the work, and approved it for publication.

## Conflict of Interest

FP serves on advisory boards for Amgen, Eisai, Eli Lilly, MSD, Novartis, Pierre-Fabre, Pfizer, and Roche; receives research funding from Astrazeneca, Eisai, and Roche; receives travel grants from Celgene and Roche; receives honoraria from Ipsen, MSD, and Takeda; all outside the submitted work. The remaining authors declare that the research was conducted in the absence of any commercial or financial relationships that could be construed as a potential conflict of interest.
